# No Pain, No Gain? Personality Associations With Awareness of Aging Depend on Arthritis

**DOI:** 10.3389/fpsyg.2022.863152

**Published:** 2022-06-09

**Authors:** Victoria J. Dunsmore, Shevaun D. Neupert

**Affiliations:** Department of Psychology, North Carolina State University, Raleigh, NC, United States

**Keywords:** attitudes toward own aging, awareness of aging, awareness of age-related change, personality, arthritis

## Abstract

**Background:**

Awareness of aging brings to light one’s own perceived behavioral, physical, and cognitive changes associated with getting older. Personality and physical illness are each related to two components of awareness of aging: attitudes toward own aging (ATOA), and awareness of age-related changes (AARC). Here, we move beyond main effects to examine how personality and arthritis interact with respect to awareness of aging.

**Materials and Methods:**

296 participants (*M* age = 64.67, *SD* = 4.36, Range = 60–90 years, 49.7% women) completed online self-report questionnaires of personality, arthritis, ATOA, and AARC gains and losses.

**Results:**

We ran three hierarchical multiple regression models to test how personality traits and arthritis interacted to predict ATOA, AARC gains, and AARC losses, respectively. Higher extraversion was related to positive ATOA, and higher openness was related to positive ATOA and more AARC gains. Higher neuroticism was related to negative ATOA, more AARC losses, and less AARC gains. We found a main effect for arthritis, where those with arthritis reported more AARC losses. Lastly, we found a significant interaction between arthritis and agreeableness when predicting AARC gains. Among those with low agreeableness, people with arthritis reported significantly more AARC gains compared to those without arthritis.

**Conclusion:**

Personality and arthritis are each important for awareness of aging. Overall, our study suggests that for those with arthritis, it is especially important to consider behavioral and cognitive factors related to agreeableness, as they may be important means of promoting positive views on aging.

## Introduction

Awareness of aging is a theoretical framework that views aging as an integral psychological process involving subjective interpretations and self-knowledge of changes as a result of getting older (Diehl et al., 2015). It was brought forth, in part, to promote more research on successful aging by examining health promotion and understanding, and to improve attitudes toward the aging process (Diehl et al., 2014). Awareness of aging is viewed as an umbrella term for various concepts involving perceptions of aging. Diehl et al. (2015) examined two foci that can determine this experience: the age-related “feedback” someone elicits from their social environment (e.g., outwardly visible cues for social judgment), and changes in functioning and/or behavior that are perceived from the individual. For the current investigation, we focused on the concepts within awareness of aging that stress the first-person perspective by examining awareness of age-related changes (AARC) which includes gains and losses, and attitudes toward own aging (ATOA). ATOA and AARC are key features of Awareness of Aging. ATOA is conceptualized as implicit, formed preconsciously but can be recalled explicitly, and tends to affect behavior in many areas of functioning. In contrast, AARC is conceptualized as explicit, emphasizing the importance that something has changed in ones’ life that is directly attributable to their own aging, and is tied to specific, multidimensional experiences (Diehl et al., 2014). We looked at how these outcomes are predicted by personality and arthritis.

### Awareness of Aging and Health

Awareness of age-related changes (AARC) is defined as “a person’s state of awareness that his or her behavior, level of performance, or way of experiencing life has changed as a consequence of having grown older” (Diehl and Wahl, 2010, p. 342). This concept involves one’s perceived connection between increasing chronological age and its direct influences on changes in functioning (Diehl et al., 2015). These changes can be viewed as either gains or losses and are influenced by person-level factors such as health and personality traits (Diehl and Wahl, 2010). However, the possible interactions among these individual characteristics in predicting AARC remain underexplored.

Health and AARC have strong connections, as defined by Diehl and Wahl (2010), who specified an example of an AARC loss in the health and physical functioning domain as “the loss of muscle mass and muscle strength, reduced bone mass and bone density, and problems with joints due to chronic illness conditions, such as arthritis” (p. 344). Brothers et al. (2017) examined how AARC mediated the relationship between affect and health and found that positive ATOA predicted more AARC gains which then was related to better functional health (Brothers et al., 2017). Physiological changes have strong ties to one’s subjective interpretation of their age (Diehl and Wahl, 2010). These changes tend to be perceived as losses due to increased chronological age, including loss of stamina, skin elasticity, and general strength (Diehl and Wahl, 2010). A systematic review conducted by Sabatini et al. (2020) found that poor physical well-being was significantly related to a higher level of AARC losses. Sabatini et al. (2021) also reported that higher levels of pain predicted more AARC losses when examined cross-sectionally. Perceived losses can develop from daily reminders of changes in the body, whether it be physically, socially, or psychologically (Neupert and Bellingtier, 2017). These daily reminders can be more strongly associated with negative AARC experiences if one has a chronic illness that consists of daily symptoms. For example, arthritis is a disease that is commonly associated with chronological aging, and can be characterized by daily pain, stiffness, and swelling (Mayo Clinic, 2021).

Another component of awareness of aging that has strong ties to health and personality is attitudes toward own aging (ATOA), which reflects individual as well as societal interpretations of aging (Hess, 2006). In western society, many older adults have negative thoughts toward aging, as various stereotypes exist in these cultures that aim to “prevent” or “reverse” the aging process (Kotter-Grühn, 2015). People with more negative attitudes toward their own aging (ATOA) tend to have poorer health behavior and lower objective health overall (Kotter-Grühn, 2015). It is also true that global health can be an important cross-sectional predictor of ATOA in young-old adults (Miche et al., 2014) and in middle-aged and older adults (Spuling et al., 2020). Although having more positive attitudes toward aging tends to be associated with better subjective health (Beyer et al., 2015), people who report more positive ATOA, on days when they report more age-related losses (e.g., “I am slower in my thinking”), had significant increases in their daily negative affect (Neupert and Bellingtier, 2017). This suggests that those with more positive aging attitudes could be particularly vulnerable to situations that threaten those positive views (i.e., increases in AARC losses) (Neupert and Bellingtier, 2017).

Physical health and awareness of aging concepts like AARC and ATOA are related bidirectionally, with some research looking at how awareness of aging can impact health (Kotter-Grühn, 2015; Tully-Wilson et al., 2021), and some looking at how health can impact awareness of aging (Zhang and Neupert, 2020). Regardless of the direction though, the evidence supports strong connections between health and awareness of aging concepts. Degenerative, non-preventative diseases such as arthritis, can make it challenging for some to interpret their aging in a positive light (Thorpe et al., 2014). A health-related event may modify one’s views of their own aging as a positive or negative experience (Montepare, 2009; Wurm et al., 2020). Specifically, Schönstein et al. (2021) found that older adults with rheumatism report more negative ATOA over time. Rheumatism includes all illnesses that incorporate inflammation of the joints (Mandal, 2019). The current study focused exclusively on arthritis because of its age-graded nature, and the daily symptoms accompanying arthritis may be associated with vulnerability to age-related losses. A systematic review done by Tully-Wilson et al. (2021) showed that positive attitudes were related to better health-related longitudinal outcomes, such as better subjective health.

### Awareness of Aging and Personality

The Big Five factors of personality are also important to consider when examining awareness of aging. OCEAN – openness (e.g., a need for novelty), conscientiousness (e.g., long-term planning), extraversion (e.g., preference for companionship), agreeableness (e.g., compliance), and neuroticism (e.g., low self-esteem) (McCrae and Costa, 1999), are cross-sectionally and longitudinally related to AARC (Rupprecht et al., 2019) as well as ATOA (Kornadt et al., 2019). For example, someone with high levels of neuroticism may report more negative attitudes toward aging, as neuroticism tends to consist of pessimistic attitudes (McCrae and Costa, 1999). Kornadt et al. (2019) used the Big Five to predict ATOA among older adults and found that lower neuroticism, higher conscientiousness, and higher openness predicted higher positive ATOA.

Personality is also associated with one’s awareness of age-related changes, as higher extraversion and neuroticism are associated with more AARC gains and losses, respectively (Diehl et al., 2014). When examined cross-sectionally, neuroticism was positively related to AARC losses, while openness and conscientiousness were positively related to AARC gains (Rupprecht et al., 2019). Interestingly, Rupprecht et al. (2019) also found that higher neuroticism at time 1 was related to higher AARC gains at time 2 (4.5 years later), which they speculate may reflect positive outcomes associated with high neuroticism. This association might be reflected in research examining “healthy neuroticism” as a mechanism for individuals to manage their illness (Friedman, 2000). Longitudinally, high conscientiousness was associated with decreases in AARC losses over time (Rupprecht et al., 2019). As reflected in this work, the relationship between personality and AARC can be multifaceted and complex, and understanding how these factors operate in the context of health is important to consider given their overlapping relationship. The current study moved these parallel research streams together by integrating all constructs into one study and testing the interactions between arthritis and personality.

### Present Study

The present study examines personality and arthritis simultaneously as person-level predictors of awareness of aging, which follows the awareness of aging theoretical perspective (Diehl and Wahl, 2010) and extends previous work which has not examined these concepts simultaneously in the same model. Objective age-related changes, including sensory functioning and physical strength, tend to be subjectively represented (Diehl and Wahl, 2010); therefore, we focused our study on arthritis as it is an age-graded illness and can involve daily experiences of pain. We expected that our main effects would be consistent with past research and focused our efforts on exploring the interactions between arthritis and personality on ATOA and AARC gains and losses.

## Materials and Methods

### Participants

Participants were from the MACE (Mindfulness and Anticipatory Coping Everyday; Neupert and Bellingtier, 2017, 2019; English et al., 2019) study, conducted over the course of 2016, and were recruited using Amazon’s Mechanical Turk (MTurk) online platform. Higher quality data can be acquired through a more diverse group of participants with MTurk when compared to standard community samples (Buhrmester et al., 2011). The study was conducted according to the guidelines of the Declaration of Helsinki and approved by the Institutional Review Board of North Carolina State University (protocol #6517, 28 April, 2016). Only participants in the United States who had not previously been diagnosed with dementia or mild cognitive impairment could participate. A total of 296 participants between 60 and 90 years of age (*M* = 64.67, *SD* = 4.36) were included in the analyses. Participants were majority White (83.8%) and married (44.6%) with at least a Bachelor’s degree (23.8%), and reported an individual income averaging between $50,000 and $59,000. Gender was evenly distributed between men (50.3%) and women (49.7%).

### Procedures

Participants were recruited through Amazon’s Mechanical Turk (MTurk). Researchers posted a Human Intelligence Task (HIT) with a link to the survey. If participants indicated that they had dementia or mild cognitive impairment, they were not allowed to proceed. For our analyses, we extracted the data from participants who were 60 years of age and older, who responded to the survey, and who satisfied the requirement of living in the United States.

After clicking the survey link, participants were directed to Qualtrics which showed the informed consent form. After consenting, participants were directed to the measures. Upon completion, they were provided an MTurk validation code and compensated $1. After data collection, researchers checked to make sure participants met all criteria as indicated by the self-report survey. There were no missing data on any of the study measures for the current study.

### Instruments

#### Personality

We used the Revised Midlife Development Inventory (MIDI) Personality Scales consisting of 31 trait adjectives answered on a 1 (a lot) to 4 (not at all) scale (Lachman and Weaver, 1997, 2005). We used the Big Five scales for this study which were openness, (seven items, e.g., creative, curious, Lachman and Weaver, 1997: α = 0.77; present study: α = 0.76); conscientiousness, (five items, e.g., responsible, organized, Lachman and Weaver, 1997: α = 0.58; present study: α = 0.81); extraversion, (five items, e.g., outgoing, lively, Lachman and Weaver, 1997: α = 0.78; present study: α = 0.78); agreeableness, (five items, e.g., helpful, warm, Lachman and Weaver, 1997: α = 0.80; present study: α = 0.88); and neuroticism, (four items, e.g., moody, nervous, Lachman and Weaver, 1997: α = 0.74; present study: α = 0.78).

#### Arthritis

The MIDUS chronic conditions checklist assessed chronic health problems within the last 12 months (MIDUS I and II; Brim et al., 1996; Ryff et al., 2006). For the purposes of the current study, we focused on the arthritis item. This was assessed by asking whether the participant had been treated for, or experienced, arthritis to which they responded with a “yes” (1) or “no” (0). A total of 100 participants out of our 296 participants reported being treated for, or experiencing, arthritis.

#### Attitudes Toward Own Aging

Aging attitudes were recorded using the ATOA subscale from the Philadelphia Geriatric Center Morale Scale (Lawton, 1975: α = 0.81; present study: α = 0.77). There were five items including one that states, “I am as happy now as I was when I was younger.” These statements were scored on a 5-point Likert scale from 1 (does not apply to me) to 5 (applies very well). Higher average scores indicated more positive attitudes toward aging.

#### Awareness of Age-Related Changes

Awareness of age-related changes was examined using the short 20-item version of the AARC Questionnaire (AARC-20; Brothers et al., 2017, gains: α = 0.96, losses: α = 0.96; present study: gains: α = 0.85, losses: α = 0.90) to capture age-related gains (e.g., “With my increasing age, I realize that I have more freedom to live my days the way I want”), and age-related losses (e.g., “With my increasing age, I realize that I have to limit my activities”). These items were assessed on a 5-point Likert scale and sum scores for each subscale were created, in line with past work (Brothers et al., 2017). These sum scores could range from 10 (low levels) to 50 (high levels). The sum scores for each subscale reflected either greater perceptions of age-related gains or losses.

#### Covariates

Gender (0 = male, 1 = female) and continuous age were used as covariates in all models.

### Data Analysis

All analyses were undertaken with SPSS (IBM Corp, 2021; Version 25). Prior to conducting hierarchical multiple regression models for each awareness of aging outcome, we ensured that the data met the assumptions for homogeneity of variance, linearity, and normal distributions. Following recommendations of Field (2015), we plotted standardized residuals (*x*-axis) with standardized predicted values (*y*-axis) to test for homogeneity of variance and linearity. The results of all three graphs were a random array of dots with no clear pattern to them, suggesting that the assumptions of homogeneity of variance and linearity were met. We verified the assumption of a normal distribution using guidance from Westfall (2014) regarding kurtosis (all values less than 3) and Pearson’s Coefficient of Skewness #2 (median) (Pearson, 1895), regarding skewness (values between −0.5 and +0.5). All variables met the criteria for mesokurtic distributions and only agreeableness (−0.52) was negatively skewed. The histograms and Normal P-P plots are available at https://osf.io/k2q9n/.

We conducted three separate hierarchical regression models where we included age and gender as covariates (Step 1), main effects of personality traits and arthritis (Step 2), and the interactions of each personality trait and arthritis (Step 3) to predict ATOA (Model 1), AARC losses (Model 2), and AARC gains (Model 3). VIF was larger than Tolerance for each model, indicating possible multicollinearity among predictors. There was no evidence of influential case bias, as the Cook’s *D* values for AARC-L, AARC-G, and ATOA are all less than one. There was also no evidence of autocorrelation among residuals as the Durbin-Watson statistic for AARC-L, AARC-G, and ATOA is, respectively, 1.86, 1.86, and 1.91. The personality traits were centered to reduce multicollinearity prior to creating the interaction terms by subtracting the sample mean. Significant interactions were decomposed using Preacher’s online calculator (Preacher et al., 2006) for simple slopes and tests of contrasts.

## Results

We calculated the descriptive information and intercorrelations among all study variables, which are presented in Table 1. We conducted Pearson correlations among the continuous variables meeting criteria for interval or ratio level of measurement. The linearity of these relationships was verified in scatterplots. There was no evidence of curvilinear associations. We used point biserial correlations for the associations between arthritis and continuous variables. To address the skewness of agreeableness, we conducted a square root transformation (Field, 2015) and then ran regression models with the transformed score and again with the non-transformed (i.e., original metric) score. The pattern of results was identical in all models; therefore, we report the non-transformed results. Results with the transformed score can be found at https://osf.io/k2q9n/. The means and standard deviations of ATOA and AARC are in line with past work (see Table 1). In the present study ATOA is moderately correlated with AARC gains and losses, but AARC gains, and losses appear to have minimal overlap with each other. Arthritis was associated with more AARC losses and lower ATOA but was not associated with AARC gains. As expected, there was overlap among each of the personality traits, but each personality trait was significantly correlated with AARC gains, AARC losses, and ATOA. Women reported more arthritis, higher conscientiousness, higher agreeableness, and more AARC gains compared to men.

**TABLE 1:** Descriptive statistics and correlations for study variables (*n* = 296).

Variable	*M*	*SD*	1	2	3	4	5	6	7	8	9	10
1. Arthritis	0.34	0.47	−									
2. OPEN	3.0	0.6	0.04	−								
3. CONSC	3.3	0.6	0.04	0.55*	−							
4. EXTRAV	2.9	0.7	−0.06	0.63*	0.55*	−						
5. AGREE	3.3	0.7	0.07	0.56*	0.59*	0.60*	−					
6. NEUROT	2.1	0.7	0.04	−0.33*	−0.45*	−0.42*	−0.28*	−				
7. AARC-G	35.8	8.0	0.07	0.44*	0.48*	0.40*	0.49*	−0.32*	−			
8. AARC-L	23.7	8.0	0.17*	−0.32*	−0.35*	−0.34*	−0.22*	0.46*	−0.15*	−		
9. ATOA	3.3	0.9	−0.11*	0.37*	0.30*	0.41*	0.25*	−0.47*	0.40*	−0.63*	−	
10. Female^a^	49.70%	−	^b^	0.03	0.23*	0.08	0.28*	0.10	0.24*	0.06	−0.04	−
11. Age	64.7	4.4	0.07	−0.06	−0.01	0.01	−0.05	−0.09	0.02	0.04	0.02	−0.004
OPEN^c^	3.0	0.5										
CONSC^c^	3.4	0.5										
EXTRAV^c^	3.2	0.6										
CONSC^c^	3.4	0.5										
AGREE^c^	3.5	0.5										
NEUROT^c^	2.3	0.7										
ATOA^d^	3.8	1.3										
AARC-G^e^	37.7	7.1										
AARC-L^e^	22.5	8.3										

*p < 0.01.

*OPEN, openness; CONSC, conscientiousness; EXTRAV, extraversion; AGREE, agreeableness; NEUROT, neuroticism; AARC-G, awareness of age-related change-gains; AARC-L, awareness of age-related change-losses; ATOA, attitudes toward own aging.*

*^a^Positive correlations with female indicate that women scored higher on the measure.*

*^b^The association between gender and arthritis was calculated using a Phi correlation, (Φ = 0.12, p = 0.04).*

*^c^Subscales from MIDI: Midlife Development Inventory (Lachman and Weaver, 1997).*

*^d^Kavirajan et al. (2011).*

*^e^Neupert and Bellingtier (2017).*

### Main Effects

#### Attitudes Toward Own Aging

In Step 2 (Model 1, Table 2), which includes all main effects, the model explained 30% of the variance in ATOA (*R*^2^ = 0.30, *F*(8,287) = 15.34, *p* < 0.001). We found that higher openness (β = 0.19, *p* = 0.007) and higher extraversion (β = −0.09, *p* = 0.017) were associated with more positive ATOA, and higher neuroticism (β = −0.36, *p* < 0.001) was associated with more negative ATOA. However, we did not find significant effects of conscientiousness (β = −0.04, *p* = 0.595), agreeableness (β = −0.04, *p* = 0.580), or arthritis (β = 0.17, *p* = 0.065) on ATOA.

**TABLE 2:** Hierarchical regression for ATOA, AARC losses, and AARC gains.

	Model 1: *ATOA*			Model 2: *AARC losses*			Model 3: *AARC gains*		
Variable	*B*	*SE* B	β	*B*	*SE B*	β	*B*	*SE B*	β
**Step 1**									
Constant	3.06**	0.75		18.91*	6.97		31.97**	6.73	
Gender	−0.07	0.10	−0.04	0.96	0.93	0.06	3.42**	0.84	0.22
Age	0.01	0.01	0.02	0.07	0.11	0.04	0.04	0.04	0.06
*R* ^2^	0.002			0.01			0.06**		
**Step 2**									
Constant	3.12**	0.78		19.41*	7.33		13.44	6.95	
Gender	0.01	0.09	0.01	0.53	0.88	0.03	2.22**	0.79	0.14
Age	0.001	0.01	0.01	0.10	0.09	0.05	−0.01	0.04	−0.02
Arthritis	−0.17	0.09	0.17	2.57**	0.86	0.15	1.05	0.80	0.06
Openness	0.26**	0.10	0.19	−1.60	0.92	−0.12	1.71*	0.83	0.14
Conscientiousness	−0.05	0.10	−0.04	−1.73	0.92	−0.13	1.67*	0.84	0.14
Extraversion	0.22*	0.09	−0.09	−0.81	0.86	0.15	−0.23	0.79	−0.02
Agreeableness	−0.05	0.09	−0.04	0.57	0.82	0.05	2.31**	0.76	0.21
Neuroticism	−0.43**	0.07	−0.36	3.79**	0.66	0.34	−2.17**	0.61	−0.19
Δ*R*^2^	0.30**			0.28**			0.29**		
**Step 3**									
Constant	3.07**	0.83		17.81*	7.76		15.57*	7.24	
Gender	−0.003	0.10	−0.002	0.34	0.89	0.02	2.02*	0.78	0.13
Age	−0.001	0.01	−0.003	0.07	0.09	0.04	−0.01	0.03	−0.01
Arthritis	−0.17	0.09	−0.09	2.66**	0.87	0.16	1.01	0.79	0.06
Openness	0.28*	0.12	0.20	−1.21	1.11	−0.09	2.23*	0.98	0.18
Conscientiousness	−0.08	0.12	−0.06	−2.25*	1.09	−0.17	1.26	0.96	0.10
Extraversion	0.26*	0.11	0.21	−0.20	1.06	−0.02	−0.94	0.93	−0.08
Agreeableness	−0.02	0.11	−0.02	0.96	1.02	0.09	4.04**	0.92	0.34
Neuroticism	−0.44**	0.09	−0.36	4.10**	0.83	0.34	−2.69**	0.74	−0.24
Arthritis*Openness	−0.07	0.21	−0.03	−1.55	2.00	−0.07	−2.32	1.79	−0.10
Arthritis*Conscientiousness	0.09	0.21	0.03	1.83	2.01	0.07	1.53	1.79	0.07
Arthritis*Extraversion	−0.14	0.20	−0.06	−1.57	1.92	−0.07	1.68	1.74	0.08
Arthritis*Agreeableness	−0.10	0.18	−0.04	−1.80	1.68	−0.08	−5.16**	1.52	−0.26
Arthritis*Neuroticism	0.02	0.15	0.01	−0.78	1.36	−0.04	1.39	1.23	0.07
Δ*R*^2^	0.01			0.01			0.03*		
*R* ^2a^	0.31			0.30			0.35		
*R*^2^ *^Adjusteda^*	0.27			0.27			0.33		

*p < 0.05, **p < 0.01.

*ΔR^2^ reflects the change in variance explained from one step to the next.*

*^a^We report the amount variance explained (R^2^, R^2Adjusted^) in ATOA, AARC losses, and AARC gains for Step 3 of each model.*

#### Awareness of Age-Related Changes Losses

In Step 2 (Model 2, Table 2), which includes all main effects, the model explained 28% of the variance in AARC losses (*R*^2^ = 0.28, *F*(8,287) = 14.17, *p* < 0.001). Higher neuroticism (β = 0.34, *p* < 0.001) was associated with more AARC losses. People with arthritis also reported more AARC losses (β = 0.15, *p* = 0.003). However, we did not find significant effects of openness (β = −0.12, *p* = 0.082), conscientiousness (β = −0.13, *p* = 0.061), extraversion (β = 0.15, *p* = 0.348), or agreeableness (β = 0.05, *p* = 0.484) on AARC losses.

#### Awareness of Age-Related Changes Gains

In Step 2 (Model 3, Table 2), which includes all main effects, the model explained 35% of the variance in AARC gains (*R*^2^ = 0.35, *F*(8,287) = 19.06, *p* < 0.001). We found that higher openness (β = 0.14, *p* = 0.007), higher conscientiousness (β = 0.14, *p* = 0.025) and higher agreeableness (β = 0.21, *p* = 0.002) were associated with more AARC gains, and higher neuroticism (β = −0.19, *p* = 0.010) was associated with less AARC gains. However, we did not find significant effects of extraversion (β = −0.02, *p* = 0.960) or arthritis (β = 0.06, *p* = 0.559) on AARC gains.

The main effects at Step 2 explained the majority of variance in ATOA, AARC losses, and AARC gains, though we did find a significant interaction in Step 3 for AARC gains.

### Personality by Arthritis Interactions

**FIGURE 1:**
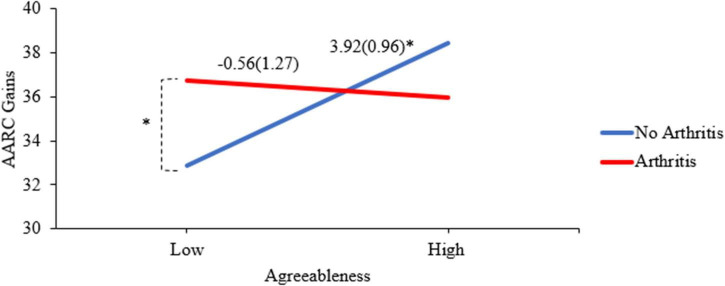
Interaction of arthritis and agreeableness predicting AARC gains. *p < 0.01. The *y*-axis values reflect predicted AARC gain scores. The slope values and (standard errors) appear above the lines for those with and without arthritis. The points were derived from predicting the values using low (Mean–1 SD) and high (Mean + 1 SD) values of agreeableness for those with and without arthritis, as recommended by Preacher et al. (2006).

We found a significant interaction between agreeableness and arthritis within the context of AARC gains (see Model 3 of Figure 1 and Table 2). The slope for those without arthritis is significantly positive (slope = 3.92, *p* < 0.001), whereas the slope for those with arthritis was not significant (slope = −0.56, *p* = 0.658). The difference in AARC gains between those with and without arthritis was significant for low agreeableness (contrast = 3.85, *p* = 0.008), but not significantly different for high agreeableness (contrast = 2.51, *p* = 0.060).

## Discussion

The current study extends previous research on relationships between personality and awareness of aging (Diehl and Wahl, 2010; Kornadt et al., 2019; Rupprecht et al., 2019), as well as physiological health and awareness of aging (Thorpe et al., 2014; Sabatini et al., 2020; Schönstein et al., 2021). Our focus on a chronic, age-graded illness (e.g., arthritis), along with each of the Big Five personality traits, allowed for an integration of previously distinct findings that focused on either personality or health with respect to how older adults experience their awareness of aging. While these awareness of aging constructs (i.e., ATOA, AARC-G, and AARC-L) have some overlap with each other, the patterns by which they are predicted by arthritis and personality differ, in line with the multidimensional nature of awareness of aging (Diehl et al., 2014), and we suggest that it is useful to keep them as separate and distinct constructs.

In line with past work, we found main effects of the Big Five on ATOA, AARC gains, and AARC losses. The Big Five has previously been shown to be associated with ATOA (Kornadt et al., 2019), AARC gains and AARC losses (Rupprecht et al., 2019), such that higher openness, agreeableness, conscientiousness, extraversion, and lower neuroticism, are each related to positive ATOA, lower AARC losses, and higher AARC gains (but see opposite effect for neuroticism and AARC gains in Rupprecht et al., 2019). However, the main effect of conscientiousness on AARC gains should be interpreted with caution because it is no longer significant when interactions are included. Our results are consistent with many previous findings, but the relationship we found between neuroticism and AARC gains was negative (i.e., more neuroticism was related to less AARC gains), while Rupprecht et al. (2019) found a positive relationship between neuroticism at time 1 and AARC gains 4.5 years later. It is possible that the positive effects of “healthy neuroticism” (Friedman, 2000) may need time to build and accumulate to show effects.

Health has also been associated with these three awareness of aging concepts, as poor health is related to negative ATOA, lower AARC gains, and higher AARC losses (Diehl and Wahl, 2010; Kotter-Grühn, 2015; Brothers et al., 2017; Neupert and Bellingtier, 2017). We found a significant main effect of arthritis on AARC losses, such that arthritis presence was related to more AARC losses. This finding extends past work which has focused on poor health more broadly; here we specifically target the age-graded chronic condition of arthritis. It is possible that poor health, broadly defined, is important for AARC gains and ATOA, which would explain why we did not find main effects of arthritis in these models.

### Agreeableness, Arthritis, and Awareness of Age-Related Changes

We found evidence of an interaction between agreeableness and arthritis to predict AARC gains, such that agreeableness was positively related to AARC gains for those without arthritis, but not for those with arthritis. We also found that among those with low agreeableness, those with arthritis reported more AARC gains than those without, reflecting how health differences in awareness of age-related gains may be restricted to a specific level of a personality trait. While previous research has connected poor health to more AARC losses (Diehl and Wahl, 2010) and less AARC gains (Brothers et al., 2017), as well as high agreeableness to low AARC losses (Rupprecht et al., 2019), this was the first study to bring them together in the same model and focus on the age-graded chronic condition of arthritis. Agreeableness is characterized by compliance, willingness to defer to others, and belief in cooperation (McCrae and Costa, 1999), which is positively associated with AARC gains in older adults without arthritis. For older adults with arthritis, however, low levels of agreeableness, perhaps indicating a tendency to advocate for oneself, is associated with higher AARC gains compared to adults without arthritis. The CDC’s Arthritis Management Program (Centers for Disease Control and Prevention, 2019) emphasizes the importance of self-management strategies and activities to play an active role in controlling one’s arthritis. Further, low agreeableness is associated with a more active decision-making style with respect to healthcare decision-making (Flynn and Smith, 2007). Our result extends previous work showing that high agreeableness may not always be beneficial, especially in financial (Matz and Gladstone, 2020) or healthcare contexts (Flynn and Smith, 2007).

### Limitations and Future Directions

This study was crucial to establish the interconnectedness of personality and physical health on the awareness of aging constructs of AARC and ATOA, but some limitations should be noted. We acknowledge that of our 15 interactions, only one was significant. We were unable to thoroughly investigate types of arthritis, as well as various health behaviors, which may be associated with how older adults have adjusted to their arthritis and how that can relate to one’s awareness of aging. A benefit of self-reporting arthritis is that it may indicate participant views of not only their actual health but the symptoms they may subjectively feel impacting their life, as discussed by Smith (2007). Future work on arthritis may benefit from including the type, severity, and duration. We further acknowledge the multicollinearity between the personality traits, which may lead to biased estimates of regression coefficients. Our estimates are limited to the unique contribution of each personality trait.

Our sample was exclusively drawn from the United States, so future work should include a more globally diverse participant pool to examine cultural similarities and differences with respect to predictors and consequences of awareness of aging. Some research suggests that the stereotypes that accompany aging, and most cultures’ views on the aging process, tend to be negative (Kotter-Grühn, 2015), but the health and personality precursors of these processes may differ.

Although the behaviors associated with personality are considered somewhat stable, symptoms of arthritis may fluctuate over time, which could then lead to varying changes in AARC and ATOA. As this study was cross-sectional, we are unable to make causal statements and acknowledge that AARC and ATOA may be antecedents of health-related outcomes and personality, reflecting the bidirectional nature of these relationships (Kotter-Grühn, 2015; Zhang and Neupert, 2020; Tully-Wilson et al., 2021). Future research could examine within-person variability in AARC and ATOA as they relate to personality and health. We suggest that future research utilize a micro-longitudinal design, repeatedly capturing awareness of aging, health symptoms, and health behaviors, to understand how personality drives and is driven by behaviors and awareness of aging. Specifically, we suggest including daily life events as proximal predictors and perceptions of control beliefs as distal predictors of AARC, in line with Diehl and Wahl’s (2010) model. In addition, we suggest that future studies could incorporate cultural influences as predictors of ATOA, in line with Diehl et al.’s (2014) Awareness of Aging framework. While some work has taken a macro-longitudinal view over years in the relationship between personality or health and awareness of aging (Kornadt et al., 2019; Rupprecht et al., 2019; Schönstein et al., 2021), micro-longitudinal examinations can account for the daily nuances of health behaviors and arthritis symptomology.

## Conclusion

Limitations notwithstanding, it was paramount to establish the potential interactive relationships between arthritis and personality, and how these interactions were related to awareness of age-related changes and attitudes toward own aging. Our results showed that normative age-related health issues and personality traits are associated with important aspects of aging, specifically awareness of age-related gains. To promote successful aging, these factors must be considered. Although many older adults may see an age-graded illness like arthritis as a negative experience, some personality traits may serve as a protective factor in promoting more awareness of age-related gains. For example, various age-graded illnesses may become more prevalent with age (e.g., cancer, cardiovascular disease, and osteoarthritis) (Jaul and Barron, 2017). The implications poor health has on ATOA and AARC, combined with our results highlighting the importance of individual differences in personality, suggests that personality interventions could be a fruitful avenue to pursue to combat poor outcomes. While our study was cross-sectional and cannot clearly show changes across time in health or personality, we suggest that future research should take this approach in order to understand whether personality interventions may be beneficial. Mroczek (2014) asserted that personality is plastic, where healthy traits can be promoted for various personality types. An integrative review from Chapman et al. (2014) also found that changes to basic personality processes (e.g., increasing self-efficacy in chronic disease patients with maladaptive personality types) could affect changes at the trait level, which could then help improve health among targeted populations. Future research could examine interventions for people with arthritis to improve behaviors associated with personality traits. For example, promoting advocacy (i.e., lower agreeableness), may help promote awareness of age-related gains for older adults with arthritis. These findings highlight the importance of considering how one’s unique personality profile relates to subjective experiences of aging, as modified by an age-graded condition, such as arthritis.

## Data Availability Statement

The datasets presented in this article are not readily available because of IRB restrictions. Requests to access the datasets should be directed to VD, vdunsmo@ncsu.edu. Analytic code is available upon request.

## Ethics Statement

The studies involving human participants were reviewed and approved by the Institutional Review Board of North Carolina State University (protocol #6517, 28 April, 2016). The patients/participants provided their written informed consent to participate in this study.

## Author Contributions

VD and SN: conceptualization. SN: methodology, data curation, writing—review and editing, supervision, project administration, and funding acquisition. VD: formal analysis, writing—original draft preparation, and visualization. Both authors read and agreed to the published version of the manuscript.

## Conflict of Interest

The authors declare that the research was conducted in the absence of any commercial or financial relationships that could be construed as a potential conflict of interest.

## Publisher’s Note

All claims expressed in this article are solely those of the authors and do not necessarily represent those of their affiliated organizations, or those of the publisher, the editors and the reviewers. Any product that may be evaluated in this article, or claim that may be made by its manufacturer, is not guaranteed or endorsed by the publisher.
